# Enumeration of minimal stoichiometric precursor sets in metabolic networks

**DOI:** 10.1186/s13015-016-0087-3

**Published:** 2016-09-19

**Authors:** Ricardo Andrade, Martin Wannagat, Cecilia C. Klein, Vicente Acuña, Alberto Marchetti-Spaccamela, Paulo V. Milreu, Leen Stougie, Marie-France Sagot

**Affiliations:** 1 Erable team, INRIA Grenoble Rhône-Alpes, 655 Avenue de l’Europe, 38330 Montbonnot Saint-Martin, France; 2 UMR CNRS 5558, LBBE, “Biométrie et Biologie évolutive”, Université Lyon 1, 43 bd du 11 Novembre 1918, 69622 Villeurbanne, France; 3 Center for Mathematical Modeling (UMI 2807 CNRS), University of Chile, Beauchef 851, 837 0456 Santiago de Chile, Chile; 4 Sapienza University of Rome, Via Ariosto 25, 00185 Rome, Italy; 5 Applied Research Department, Tecsinapse, São Paulo, Brazil; 6 CWI, Science Park 123, 1098 XG Amsterdam, The Netherlands; 7 Vrije Universiteit Amsterdam, De Boelelaan 1105, 1081 HV Amsterdam, The Netherlands

**Keywords:** Metabolic network, Minimal precursor sets, Mixed integer linear programming

## Abstract

**Background:**

What an organism needs at least from its environment to produce a set of metabolites, e.g. target(s) of interest and/or biomass, has been called *a minimal precursor set*. Early approaches to enumerate all minimal precursor sets took into account only the topology of the metabolic network (topological precursor sets). Due to cycles and the stoichiometric values of the reactions, it is often not possible to produce the target(s) from a topological precursor set in the sense that there is no feasible flux. Although considering the stoichiometry makes the problem harder, it enables to obtain biologically reasonable precursor sets that we call *stoichiometric*. Recently a method to enumerate all minimal stoichiometric precursor sets was proposed in the literature. The relationship between topological and stoichiometric precursor sets had however not yet been studied.

**Results:**

Such relationship between topological and stoichiometric precursor sets is highlighted. We also present two algorithms that enumerate all minimal stoichiometric precursor sets. The first one is of theoretical interest only and is based on the above mentioned relationship. The second approach solves a series of mixed integer linear programming problems. We compared the computed minimal precursor sets to experimentally obtained growth media of several *Escherichia coli* strains using genome-scale metabolic networks.

**Conclusions:**

The results show that the second approach efficiently enumerates minimal precursor sets taking stoichiometry into account, and allows for broad in silico studies of strains or species interactions that may help to understand e.g. pathotype and niche-specific metabolic capabilities. sasita is written in Java, uses cplex as LP solver and can be downloaded together with all networks and input files used in this paper at http://www.sasita.gforge.inria.fr.

**Electronic supplementary material:**

The online version of this article (doi:10.1186/s13015-016-0087-3) contains supplementary material, which is available to authorized users.

## Background

The question of which metabolites an organism needs from its environment (henceforth called the *sources*) in order to grow or to produce a given set of metabolites (henceforth called the *targets*) is crucial for both fundamental and applied reasons. This indeed enables to define the growth conditions of organisms in the laboratory, as well as the minimal media necessary for the production of compounds of biotechnological interest (for instance, ethanol). More recently, great interest in establishing which nutrients are exchanged among different organisms in communities such as present in the human gut has also been raised by the interest to develop new strategies for fighting infection that rely on the use of probiotics instead of antibiotics [1]. However the latter requires that: (1) such exchanges are computed in a very efficient way in genome-scale metabolic networks; (2) all possible minimal sets of sources are identified for a given target set of interest in order to fully understand the interactions that may take place among the organisms in a community, as well as the alternative niches that may with time develop for some such organisms.

Early attempts at enumerating all *minimal precursor sets* (minimal sets of sources) were based only on topology (henceforth called *topological precursor sets*). Stoichiometry was thus not taken into account, leading to possibly many unfeasible solutions [2–5]. The algorithm of Romero and Karp was based on a backtrack traversing of the metabolic graph from the target compounds to the seeds while Handorf et al. tested the reachability of the target from a heuristically defined collection of sets of sources. Neither enumerated all minimal precursor sets. Cycles, although omnipresent in metabolic networks (e.g. Krebs cycle), were not included until the method of Cottret et al.  [4]. However, the latter algorithm could be applied only to small networks due to a high memory requirement; subsequently, Acuña et al. [5] allowed the enumeration of all minimal precursor sets of networks of about 1000 reactions. The authors also pointed out that the enumeration of precursor sets and of precursor cut sets could be done simultaneously in quasi-polynomial total time. Precursor cut sets are a set of sources such that, if they are eliminated, then the target set of interest can no longer be produced by any combination of the remaining sources.

The approach of Zarecki et al. [6] takes stoichiometry into account and consists of two steps. First, the size of a set of sources of minimal cardinality that allows the production of a target is determined solving a mixed integer linear programming problem. In a second step, the authors identify a single set of sources of the determined size such that the sum of the molecular weight of the compounds is minimal.

To our knowledge, there are two algorithms that attempt to enumerate all minimal precursor sets with stoichiometry (henceforth called *stoichiometric precursor sets*) [7, 8].

Imieliński et al. [7] propose a method that first enumerates all extreme semipositive conservation relations (ESCR), that is the extreme rays of the cone defined by the transposed stoichiometric matrix. The precursor sets are then obtained by the enumeration of hitting sets of the ESCRs. As the authors state, this approach is impractical for genome-scale metabolic networks since it is impossible to enumerate all ESCRs with the current algorithms [7]. Consequently, a method is proposed that enumerates a subset of the ESCRs (those that do not contain water) to obtain (via hitting sets) minimal precursor sets that contain water. These solutions are physiologically minimal (all media contains water), but not necessarily the theoretically minimal.

**Fig. 1:**
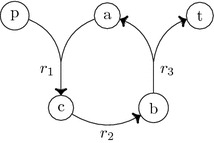
Network with one source *p* and one target *t* illustrating the difference between the two models used by Eker et al. [8], and the limitation of the machinery-duplicating model. The source *p* is a precursor set for the production of the target if the steady-state model is assumed. In this toy example, the target can not be produced following the machinery-duplicating model

The method of Eker et al. [8] is based on logical and linear constraint solving and on computational boolean algebra. The authors formulated two different constraint models, that were called *steady-state* and *machinery-duplicating*. Their steady-state model requires a non-negative net production of all compounds that are on the path from the precursors to the target. Observe that the term steady-state is usually used to denote a slightly different model where all compounds that are on the path from the precursors to the target cannot accumulate. Their machinery-duplicating model is more restrictive as it requires a strict positive net production of these compounds. Notice that a set of sources that allows the production of the target(s) in the machinery-duplicating model, allows also the production of the same target(s) in the steady-state model. A toy example illustrates the difference between the two models (see Fig. 1). In this network, we have a source (*p*), a target (*t*), internal compounds (*a*, *b*, *c*), and three reactions ($r_{1:} \,p + a \rightarrow c, r_{2:}\, c \rightarrow b, r_{3:}\, b \rightarrow a + t$). Following their steady-state model, the source *p* is a precursor of *t*. The compounds *a*, *b*, and *c* have a zero net production when we assign a positive flux value 1 to each reaction. In the machinery-duplicating model, there is no precursor set that enables to produce *t*: indeed, no flux would fulfil the condition of a strict positive net production of *a*, *b*, and *c*. However, this type of cycle resembles the Krebs cycle that plays an essential role in the production of energy in aerobic organisms. To reveal the similarity between the toy example and the Krebs cycle, let the compound *a* take the role of oxaloacetate (which is regenerated through the Krebs cycle), the source *p* feed the cycle as acetyl-CoA, the compound *b* be any compound on the Krebs cycle such as e.g. citrate or succinate, and the target *t* any by-product of the Krebs cycle such as NADH or carbon dioxide. We argue that the machinery-duplicating model is too restrictive as therein cycles of the type shown in Fig. 1 are not captured.

An approach widely used in flux-balance analysis (FBA) [9] to model an organism’s growth condition is to include a so-called biomass reaction, that consumes in the right amounts every compound needed for a cell to duplicate. Such a reaction has a single product, an artificial compound (representing the duplicated cell) that can be modelled as a target compound in our enumeration approach: a cell can grow and duplicate if it can produce this target compound.

The objective of this paper is twofold. On the theoretical level, we show the relationship between topological and stoichiometric precursor sets and we discuss some complexity results. On the methodological level, we provide two algorithms that enumerate all minimal stoichiometric precursor sets. The first one is based on the above mentioned relationship between topological and stoichiometric precursor sets. Although interesting in terms of theory, it however is not efficient in practice. The second approach, called sasita, uses a similar approach as in [10–12]. Therein the authors enumerate reaction subsets solving recursively mixed integer linear programming (MILP) problems. The reaction subsets correspond to alternate flux distributions to obtain an identical value of the objective function [10], the *k*-shortest elementary flux modes (EFM) [11] or the smallest minimal cut sets (MCS) [12]. All approaches enumerate minimal reaction sets. Here we consider minimality of the set of compounds.

sasita enables to enumerate all stoichiometrically feasible minimal precursor sets in both models, *steady-state* and *machinery-duplicating*, as in [8]. A natural question is to compare our results with those obtained in [8]. Unfortunately, the algorithm of Eker et al. [8] was not publicly available for testing. We nevertheless tried to reproduce the authors’ results by incorporating their definitions in our method, but we were unable to obtain the same results, even using the same *Escherichia coli* network they made available. More surprisingly, we found a precursor set that is minimal with respect to the solutions found by Eker et al.

In the enumeration of minimal precursor sets for a given target set, Eker et al. [8] are able to work with genome-scale metabolic networks and are exhaustive, but their method is very time and memory consuming. The authors indeed indicate that it required 3 days of execution on a 24-core (with Hyper threading) 2.67 GHz Intel X5650 Xeon CPU-model processor, using the machinery-duplicating model on an *Escherichia coli* network composed of 2314 unidirectional reactions (no reversible reactions) of which 388 were transport reactions, to enumerate 787 solutions.

We show that we can apply sasita on big networks like the iJO1366 reconstruction of the *Escherichia coli* K-12 MG1655 with 3646 reactions and 2258 compounds.

In the "Background" section, we provide basic definitions; in particular, we extend ideas from topological precursor sets as defined in [5] in order to incorporate stoichiometry, and we discuss the relationship between topological and stoichiometric solutions. We then describe the sasita algorithm for enumerating all stoichiometrically feasible minimal precursor sets. In the "Results" section, we discuss in detail the comparison with respect to Eker’s et al. proposal. Here we observe that sasita is the first publicly available software to enumerate minimal stoichiometric precursors sets both with the steady state and the machinery duplicating model. Experiments show that sasita can be applied to large genome-scale metabolic networks and we discuss the obtained results.

## Methods

### Definitions and properties

A metabolic network is composed of a set of compounds together with the reactions that transform them. The following example represents a metabolic network with four compounds and two reactions (the values before each compound in a reaction are the *stoichiometric coefficients* of the reaction):

$$\begin{aligned} r_{1:}& \,1.0 \, c_{1} + 2.0 \, c_{2} \rightarrow 1.0 \, c_{3}, \\ r_{2:}& \,3.0 \, c_{3} \rightarrow 1.0 \, c_{4}. \end{aligned}$$

In the following, we use directed hypergraphs [13, 14] to model a metabolic network. A metabolic network is characterised by a pair $\mathcal {N}=(\mathcal {C},\mathcal {R})$, where $\mathcal {C}$ is the set of vertices (representing metabolic compounds) and $\mathcal {R}$ is a set of hyperarcs (representing metabolic reactions). All reactions are considered to be irreversible. Reversible reactions are thus split into a forward and a backward reaction. A stoichiometric matrix *S* associated to $\mathcal {N}$ is a matrix containing the stoichiometric coefficients of each reaction with the reactions in $\mathcal {R}$ as its columns and the compounds in $\mathcal {C}$ as its rows. We define $\mathcal {X}\subseteq \mathcal {C}$ as the set of *source compounds* and $\mathcal {T}\subseteq \mathcal {C}$ as the set of *target compounds*. For simplicity, we assume that sources are not produced by any reaction; it is easy to verify such condition by adding for each metabolite *x* in $\mathcal {X}$ a dummy metabolite $x'$ and a dummy reaction producing one *x* from one $x'$. Replacing *x* by their representative $x'$ in the set $\mathcal {X}$ produces an equivalent network (in terms of what a set of sources is able to produce) with the desired property (see [5]).

Topologically, a reaction $r \in \mathcal {R}$ is defined by its substrates ${\textit{S}ubs(r)}$ and its products ${\textit{P}rod(r)}$, suggesting the interpretation of a reaction as an hyperarc with ${\textit{S}ubs(r)}$ as the set of tail nodes and ${\textit{P}rod(r)}$ as the set of head nodes of a reaction *r*. In the above example, ${\textit{S}ubs(r_{1})} = \{c_{1},c_{2}\}$ and ${\textit{P}rod(r_{1})} = \{c_{3}\}$. The network $\mathcal {N}$ can then be seen as a directed hypergraph with stoichiometric coefficients associated with each hyperarc. Given a subset of reactions $F\subseteq \mathcal {R}$, we denote by ${\textit{S}ubs(F)}$ and ${\textit{P}rod(F)}$ the union of the substrates and products, respectively, of the reactions in *F*.

Given a network $\mathcal {N}$ and the sets of source $\mathcal {X}$ and target $\mathcal {T}$ compounds, we loosely define a precursor set as a set $X \subseteq \mathcal {X}$ that can *produce* all the targets of $\mathcal {T}$ using a subset of the reactions in $\mathcal {R}$. The concept of a *factory* was introduced in [5]; a *topological factory* is defined as follows:

#### **Definition 1**

A set $F \subseteq \mathcal {R}$ is a *topological factory* from $X \subseteq \mathcal {X}$ to $T \subseteq \mathcal {T}$ if $T \cup {\textit{S}ubs(F)} \subseteq {\textit{P}rod(F)} \cup X$; i.e., if *T* and every substrate of every reaction in *F* is either a source or is produced by some reaction in *F*.

A set $X \subseteq \mathcal {X}$ is a *topological precursor set* (TPS) for $\mathcal {T}$ if there exists a topological factory from *X* to $\mathcal {T}$.

Extending this definition to include stoichiometry requires that any substrate of a reaction in *F*, should be either produced at least in a same quantity by one or more reactions also in *F*, or the substrate should be a source compound. Observe that, if the flux vector $v \in \mathbb {R}^{|\mathcal {R}|}$ denotes the flux of every reaction in the network per time unit, then $Sv\in \mathbb {R}^{|\mathcal {C}|}$ is the vector of net production of all compounds in the network for the flux *v*. Furthermore, $(Sv)_{A}$ specifies the net production of the compounds in a set *A*.

#### **Definition 2**

A **stoichiometric factory** (S-factory) from $X \subseteq \mathcal {X}$ to $T \subseteq \mathcal {T}$ is a set $F \subseteq \mathcal {R}$, such that there exists a flux vector $v \ge 0$ satisfying:

1. $$v_{i} \left\{ \begin{array}{ll} > 0 & \quad i \in F\\ = 0 & \quad \text {otherwise,} \end{array} \right.$$
2. $$(Sv)_{\mathcal {C}\setminus X} \ge 0$$,
3. $$(Sv)_{T} > 0$$.

A S-factory from *X* to *T* is **minimal** if it does not contain any other S-factory from *X* to *T*.

It is possible to adapt Definition 2 to the steady-state assumption by replacing the greater-than-or-equal sign by the equal sign in the second constraint. Thus, the steady-state constraint is put on the compounds in $\mathcal {C}$ that are neither in *X* nor in $\mathcal {T}$.

#### **Definition 3**

A set $X \subseteq \mathcal {X}$ is a **stoichiometric precursor set** (SPS) **of ***T* if there exists a S-factory from *X* to *T*. A SPS of *T* is **minimal** if it does not contain any other SPS of *T*.

The following **facts** summarise the main differences between TPSs and SPSs:

1. *Every S-factory is a topological factory. Every SPS is also a TPS.*
2. *Not every topological factory is a S-factory. Not every TPS is a SPS.*

Given these facts, it is clear that any S-factory always contains a topological factory. A natural question that arises is whether we can decompose an S-factory into a set of topological factories. We show that this is not true:

3. *There exist minimal S-factories which are not the union of minimal topological factories.*
4. *There exist minimal SPSs which do not consist of a union of minimal TPSs.*

**Fig. 2:**
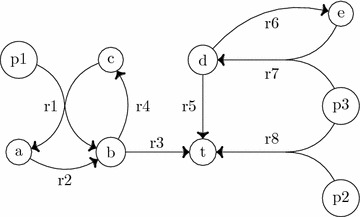
Illustration of facts 2–4. The stoichiometric values are all equal to one. There are two minimal TPSs: $\{p1\}$ (obtained from the topological factory $\{r_1,\ r_3,\ r_4\}$), and $\{p3\}$ (obtained from the topological factory $\{r_7,\ r_6,\ r_5\}$). The source *p*2 does not take part of a minimal topological factory because its consumption involves the consumption of the source *p*3, which forms already a minimal TPS. There are two minimal SPSs: $\{p1\}$ (obtained from the stoichiometric factory $\{r_1,\ r_2,\ r_3,\ r_4\}$), and $\{p2, p3\}$ (obtained from the stoichiometric factory $\{r_8\}$)

The first fact is a direct consequence of the definitions of SPS and TPS. The remaining facts are illustrated using Fig. 2 that has two minimal TPSs ($\{p1\}$ and $\{p3\}$), and two minimal SPSs ($\{p1\}$ and $\{p2, p3\}$) of the target set $\{t\}$. Observe that $\{p3\}$ is a (minimal) TPS but is not a SPS (fact 2). The minimal stoichiometric factory from *p*1 to *t* consists in the set of reactions *r*1, *r*2, *r*3, and *r*4, while the minimal topological factory from *p*1 to *t* does not contain the reaction *r*2 from *a* to *b* (fact 3). The minimal SPSs $\{p2, p3\}$ cannot be obtained as combinations of any minimal TPSs (fact 4). Figure 2 gives an intuition about the facts; similar characteristics can be found in real metabolic networks as well.

Figure 2 shows an example where it is indeed not possible to obtain the minimal S-factory that contains *r*1, *r*2, *r*3, and *r*4 from minimal topological factories. However, it is true that every minimal S-factory is a union of minimal topological factories of the many-to-one transformed network defined as follows:

#### **Definition 4**

Given $\mathcal {N}=(\mathcal {C},\mathcal {R})$, the *many-to-one transformation* of $\mathcal {N}$ is the metabolic network $\Psi (\mathcal {N})=(\mathcal {C},\Psi (\mathcal {R}))$ such that for each reaction $r\in \mathcal {R}$ and for each metabolite $a\in {\textit{P}rod(r)}$, there is a reaction $r_a$ in $\Psi (\mathcal {R})$ such that ${\textit{S}ubs(r_a)}={\textit{S}ubs(r)}$ and ${\textit{P}rod(r_a)}=\{a\}$.

Given a reaction $r \in \mathcal {R}$ with ${\textit{P}rod(r)}=\{a_1,\ldots ,a_k\}$, we denote by $\Psi (r)=\{r_1,\ldots ,r_k\}$ the set of *k* reactions in $\Psi (\mathcal {R})$ that correspond to the many-to-one transformation of *r*, that is, ${\textit{S}ubs(r_i)}={\textit{S}ubs(r)}$ and ${\textit{P}rod(r_i)}=\{a_i\}$. Furthermore, we extend this definition to sets of reactions, that is, if $R\subseteq \mathcal {R}$, we denote $\Psi (R)=\cup _{r\in R} \Psi (r)$.

It is clear that all minimal topological factories in $\mathcal {N}$ are also among the minimal topological factories in the many-to-one network $\Psi (\mathcal {N})$ if we would retransform the many-to-one reactions into their original hyper-reactions. However, there are additional minimal topological factories in $\Psi (\mathcal {N})$ that are not minimal in $\mathcal {N}$ after the retransformation. From this, we claim that every minimal S-factory in $\mathcal {N}$ is a union of minimal topological factories of $\Psi (\mathcal {N})$. The following definition and lemmas will provide the basis for the proof of this statement.

#### **Definition 5**

In a hypergraph, we define a (simple) path $p=(M,R)$ from *s* to *t* as a chain of different metabolites $M=(m_0,\ldots ,m_n)$ and a chain of different reactions $R=(r_1,\dots ,r_n)$ such that:

1. $$m_0=s$$ and $$m_n=t$$;
2. $$m_i \in Subs(r_{i+1}),\ i\in \{0,\ldots ,n-1\}$$;
3. $$m_i \in Prod(r_i),\ i\in \{1,\ldots ,n\}$$.

#### **Lemma 1**

*Given a minimal S-factoryHfrom *$X \in \mathcal {X}$*to*$T \in \mathcal {T}$*in the network *$\mathcal {N}$*, for every reaction *$r \in H$*, there is always at least one path in Hfrom one of the products of rto some metabolite in*$\mathcal {T}$.

#### Proof

Let us suppose without loss of generality that $|T| = 1$. We are going to prove the lemma by contradiction. Suppose that there is a reaction $r \in H$ such that ${\textit{P}rod(r)}=\left\{ p_{1},\dots ,p_{k}\right\}$, and that for all $p_{i} \in {\textit{P}rod(r)}$ there is no path from $p_{i}$ to *T* in *H*. Since there is no path to *T* in *H* that includes *r*, if $r' \in H$ is a reaction that consumes one product of *r*, then there cannot be a path to *T* that includes $r'$ (in fact if such a path exists then there is also a path that includes *r*). By repeating the same reasoning, consider the set of reactions $I_{r}$ corresponding to the reactions in *H* that consume the products of *r* and of the reactions that consume the products of those reactions and so on. Let $\overline{H}= H\setminus I_{r}$; we will argue that $\overline{H}$ remains a stoichiometric factory.

Consider *S* the stoichiometric matrix associated to *N* and *v* the positive vector associated with *H*. Removing *r* from *H* means $v_{r}=0$. Consider $\overline{v}$, a vector with the same values of *v* except for the components of *v* corresponding to the reactions of $I_{r}$ (the fluxes corresponding to the reactions in $I_{r}$) which should be zero. Since any of the reactions of $I_{r}$ lead to *T*, we have that:

$$\begin{aligned} S\overline{v}\ge 0, \quad (S\overline{v})_{T} \ge 1. \end{aligned}$$

Since $\overline{v_{k}}> 0$ for all $k \in \overline{H}$, $\overline{H}\subset H$ is a stoichiometric factory, which is a contradiction because *H* is minimal. $\square$

#### **Lemma 2**

*Given a set of reactions *$H\subseteq \Psi (\mathcal {R})$*and the set of sources *$X={\textit{S}ubs(H)}\cap \mathcal {X}$*, thenH is a minimal topological factory from Xto *$\mathcal {T}$*if and only if the two following statements are true:*

1. For every metabolite *m* in ${\textit{S}ubs(H)}\setminus X$ there is exactly one reaction in *H* that produces *m*;
2. For every metabolite *m* in ${\textit{P}rod(H)}$ there exists a path from *m* to $t\in \mathcal {T}$ contained in *H*.

#### Proof

We first prove that if *H* is a minimal topological factory from *X* to $\mathcal {T}$, then both statements (1 and 2) above hold. By definition, *H* is a topological factory from *X* to $\mathcal {T}$ if and only if any metabolite in $\mathcal {T}$ and in ${\textit{S}ubs(H)}$ is a source in *X* or is produced by some reaction in *H*. Let *m* be a metabolite in ${\textit{S}ubs(H)}\setminus X$. Then by definition there is a reaction *r* in *H* that produces *m*. Suppose however that there is another reaction $r'$ in *H* also producing *m*. Then ${\textit{P}rod(H\setminus \{r'\})}= {\textit{P}rod(H)}$. Thus, $H\setminus \{r'\}$ would still be a topological factory from *X* to $\mathcal {T}$ which contradicts the minimality and thus proves Statement 1.

Now let *m* be a metabolite in ${\textit{P}rod(H)}$. We show that there is a path from *m* to some target in $\mathcal {T}$. By contradiction, suppose that there is no such path. Consider then the following iterative process. Starting from $M=\mathcal {T}$ and $R=\emptyset$, consider all reactions $H'$ of *H* that produce the metabolites in *M*. Then add to *R* all reactions in $H'$ and to *M* all substrates of $H'$, and repeat the process until no reaction is added. Clearly, for all $m \in M$, either $m \in \mathcal {X}$ or *m* is produced by some reaction in *R*, and therefore *R* is a topological factory from *X* to $\mathcal {T}$. Since all reactions in $\mathcal {R}$ are also in *H* and *H* is a minimal topological factory, $R=H$ and *m* must have been included in *M* in some iteration. Clearly from that iteration, we can recover a path from *m* to some metabolite in $\mathcal {T}$ by going backwards in the described process and therefore Statement 2 above holds.

In order to prove the opposite implication, we first observe that, if both statements are true, then by Definition 1, the set *H* corresponds to a topological factory from *X* to $\mathcal {T}$. Therefore, we only need to show that it corresponds to a *minimal* topological factory. Let $H'\subseteq H$ be a minimal topological factory from *X* to $\mathcal {T}$. By contradiction, suppose that $H'\ne H$, then there is a reaction *r* in $H\setminus H'$. Let *a* be the product of *r*. By hypothesis, there is a path from *a* to $\mathcal {T}$ in *H*. However each reaction in the path is the only one in *H* producing the metabolites composing its products. Clearly the last reaction in the path (which produces a target) must also belong to $H'$. Thus, at some point in the path, there is a metabolite which is the product of a reaction in *H* which is not in $H'$, and is the substrate of a reaction in $H'$. There is thus a substrate of a reaction in $H'$ that is not produced by any reaction in $H'$, which is a contradiction with the fact that *H* is a topological factory from *X* to $\mathcal {T}$. Therefore, $H'=H$ and the minimality is proved. $\square$

The following theorem shows that any minimal S-factory is the union of minimal topological factories in the many-to-one network.

#### **Theorem 1**

*For any minimal S-factory *$H \subseteq \mathcal {R}$*fromXtoTin*$\mathcal {N}$*, there exists a set of minimal topological factories *$F_{1}, \ldots , F_{k}$*fromXtoTin*$\Psi (\mathcal {N})$*such that:*

1. $$F_{1}, \ldots , F_{k}\subseteq \Psi (H)$$;
2. For each reaction *r* in *H* there is $i\in \{1,\ldots ,n\}$ such that $\Psi (r)\cap F_i\ne \emptyset$.

#### Proof

From a given minimal S-factory *H*, we select a reaction $r \in H$. By Lemma 1, we know that there is a path from at least one product *m* of *r* to one target compound *t*. Clearly, there is a path $p=(M_p,R_p)$ from *m* to *t* in $\Psi (\mathcal {N})$. Since $\Psi (\mathcal {N})$ is a many-to-one network, every metabolite in $M_p$ is produced by only one reaction in $R_p$. We show that *p* can be extended to a topological factory from *X* to *t*. Starting from the set $R_0=R_p$, we consider the set of metabolites $M_0={\textit{S}ubs(R_0)}\setminus {\textit{P}rod(R_0)}$, that is, the set of substrates that are not produced by any reaction in the set. Let *c* be any metabolite in $M_0$. In the S-factory *H* in $\mathcal {N}$, there exists a reaction *h* that produces *c*. Let $h_c$ be the many-to-one reaction in $\Psi (h_c)$ that produces *c*, that is, ${\textit{P}rod(h_c)}=\{c\}$. We define $R_1=R_0\cup \{h_c\}$ as the new set of reactions and $M_1={\textit{S}ubs(R_1)}\backslash {\textit{P}rod(R_1)}$. We repeat this process defining $R_{i+1}$ and $M_{i+1}$ by choosing any metabolite in $M_i$ until $M_{i+1}$ is empty. By construction, the set of reactions $F=R_{i+1}$ satisfies the two properties of Lemma 2, and therefore *F* is a minimal topological factory from *X* to *t* contained in $\Psi (H)$. Repeating this process for every reaction $r\in H$, we obtain a set $F_{1}, \ldots , F_{k}$ of topological factories from *X* to *t* satisfying the desired properties. $\square$

**Fig. 3:**
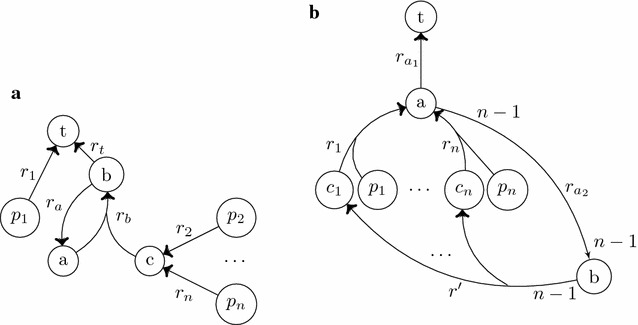
**a** A network with $\mathcal {R}= \{r_t, r_a, r_b, r_1,\dots , r_n\}$. Reaction $r_i$ with $i = 2,\dots ,n$ consumes $p_i$ and produces compound *c*. $\mathcal {T}= \{t\}$, $\mathcal {X}= \{p_1,\dots ,p_n\}$. All stoichiometric values are equal to one. There is one minimal $SPS$ ($\{p_1\}$) and *n* minimal topological factories in $\psi (\mathcal {N})$ . One contains only $\psi (r_1)$. The other minimal topological factories contain each $\{\psi (r_t), \psi (r_a), \psi (r_b)\}$ and one of the reactions in $\{\psi (r_2),\dots , \psi (r_n)\}$, respectively. **b** In this network, the set of compounds is given by $\mathcal {C}= \{a,b,t,c_1,\dots ,c_n, p_1,\dots ,p_n\}$. The compounds $p_1,\dots ,p_n$ are the sources and *t* is the target. The stoichiometric values are equal to 1 if not stated otherwise. Beside the reactions $r_{a_1}: a \rightarrow t$ and $r_{a_2}: a \rightarrow b$, there is the reaction $r'$ that consumes $n-1$ *b* and produces $\{c_1,\dots ,c_n\}$ (1 each). Furthermore, there are *n* reactions with ${\textit{S}ubs(r_i)} = \{c_i, p_i\}$ and ${\textit{P}rod(r_i)} = \{a\}$, with $i=1,\dots ,n$. The *dots* in the Figure illustrate the products $c_2,\dots ,c_{n-1}$ of $r'$ that are not shown for simplicity. The reactions $r_2,\dots ,r_{n-1}$ are not shown for the same reason. There are *n* minimal topological factories in $\psi (\mathcal {N})$, each containing the reactions $\{\psi (r_{a_1}), \psi (r_{a_2}), \psi (r')\}$ and one of the many-to-one reactions of $\{\psi (r_1),\dots ,\psi (r_n)\}$, respectively. The only minimal $SPS$ contains all sources

The theorem suggests that a straightforward idea to enumerate all $SPSs$ is to enumerate minimal topological factories in $\Psi (\mathcal {N})$ and then just build combinations thereof checking their stoichiometric feasibility in $\mathcal {N}$. The combinations can be done in the following way. We check all combinations of *k* minimal topological factories for feasibility, starting with $k=1$. Before incrementing *k*, we test if there is a minimal $SPS$ (with respect to the already obtained $SPSs$) that can be build from at least $k+1$ minimal topological factories. This however is in general not an efficient approach because (*i*) many topological factories in $\Psi (\mathcal {N})$ are not part of a $SPS$, (ii) the powerset of all topological factories in $\Psi (\mathcal {N})$ has to be built to obtain $SPSs$. Issue (*i*) is illustrated in the network of Fig. 3a. There are *n* minimal topological factories in $\Psi (\mathcal {N})$. One contains only $\psi (r_1)$. The other minimal topological factories contain each $\{\psi (r_t), \psi (r_a), \psi (r_b)\}$ and one of the reactions in $\{\psi (r_2),\dots , \psi (r_n)\}$, respectively. The only $SPS$ consists of $p_1$ and can be obtained directly from the minimal topological factories of $\Psi (\mathcal {N})$. The enumeration of the minimal topological factories that contain one of the reactions in $\{\psi (r_2),\dots , \psi (r_n)\}$ may be time consuming, for nothing since none of them yields a $SPS$. Indeed, the number of minimal topological factories in $\Psi (\mathcal {N})$ can be much higher than the number of SPSs in $\mathcal {N}$. Issue (*ii*) is depicted in Fig. 3b. There are *n* minimal topological factories in $\Psi (\mathcal {N})$. Only the combination of all *n* minimal topological factories yield a $SPS$ in $\mathcal {N}$. However, all other combinations (that can be huge for large values of *n*) have to be considered and tested for feasibility.

### Complexity

We now discuss the main complexity results for finding and enumerating $SPSs$. The next theorem shows that deciding whether a set is a $SPS$ can be done efficiently.

#### **Theorem 2**

*Given a network *$\mathcal {N}$*, a subset*$X\subseteq \mathcal {X}$*of sources and a target set*$\mathcal {T}$*, we can decide in polynomial time whetherXis a SPS for *$\mathcal {T}$.

#### Proof

We are going to show that it suffices to solve a linear optimisation problem to decide whether *X* is a SPS for $\mathcal {T}$.

Consider the network $\overline{\mathcal {N}}= (\overline{\mathcal {C}}, \overline{\mathcal {R}})$ with $\overline{\mathcal {C}}= \mathcal {C}\cup \{t\}$ and $\overline{\mathcal {R}}= \mathcal {R}\cup \{\overline{r}\}$. We add a compound *t* and a reaction $\overline{r}$ to the network $\mathcal {N}$. The reaction $\overline{r}$ is build as follows: ${\textit{S}ubs(\overline{r})} = \mathcal {T}$ and ${\textit{P}rod(\overline{r})} = \{t\}$. Also, the values of all stoichiometric coefficients of $\overline{r}$ are one. Consider now the following optimisation problem:

$$\begin{aligned} \begin{array}{cll} &{}{\displaystyle \text {Maximize}\,\,f(v) = \left( Sv\right) _{t}}\\ &{}s.t.\\ &{}\qquad \qquad \left( Sv\right) _{\mathcal {C}\setminus X} \ge 0,\\ &{}\qquad \qquad {\displaystyle v_{i} \ge 0,} &{} i=1,\dots , |\overline{\mathcal {R}}|, \end{array}\qquad \qquad \qquad \qquad {(M1)} \end{aligned}$$

where *S* represents the stoichiometric matrix of $\overline{\mathcal {N}}$.

If $v^{*}$ is a solution to M1 and $f(v^{*}) > 0$ then the support of $v^{*}$ is a stoichiometric factory from *X* to $\mathcal {T}$ and *X* is a stoichiometric precursor set for $\mathcal {T}$. $\square$

As concerns the problem of enumerating all solutions, we first observe that the proof that enumerating all minimal TPSs cannot be done in polynomial total time (that is, in the size of the input and the number of solutions) unless P = NP given in [5] can be immediately applied to show that enumerating all minimal SPSs cannot be done in polynomial total time unless P=NP. The same observation holds for enumerating all minimal cut sets (SCSs), which we define as follows:

#### **Definition 6**

A set $X \subseteq \mathcal {X}$ is a **stoichiometric cut set** (SCS) (**topological cut set** (TCS)), if $\mathcal {X}\setminus X$ is **not** a stoichiometric precursor set (topological precursor set).

We now show that the simultaneous enumeration of minimal SPSs and SCSs can be done in quasi-polynomial time. Notice that in [5], a quasi-polynomial time algorithm to simultaneously enumerate all TPSs and TCSs was presented by formulating the problem with a monotone boolean formula and then using a result of [15]. Such approach is possible even in the case of SPSs and SCSs.

#### **Theorem 3**

*The set of minimal SPSs and the set of minimal SCSs can be enumerated in total quasi-polynomial time.*

#### Proof

Define the Boolean function $f{:}\,2^\mathcal {S}\rightarrow \{0,1\}$ as $f(X)=1$ if *X* is a SPS and $f(X)=0$ otherwise. Clearly, this function is *monotone*: if $f(X)=1$ then $f(Y)=1$ for any set $Y\supseteq X$. The collection $\mathcal {P}$ of minimal SPSs is the collection of all minimal sets in $\mathcal {S}$ that evaluate to 1 and the collection $\mathcal {C}$ of minimal SCSs is the collection of all minimal sets whose complement in $\mathcal {S}$ evaluates to 0. In the context of monotone Boolean functions, minimal SPSs correspond to the *prime implicants* and minimal SCSs to the *prime implicates* of *f*. In [15], a general algorithm is proposed to jointly enumerate prime implicants and prime implicates of any Boolean function. The algorithm and time analysis are rather technical and we only give a brief description of the incremental algorithm applied to our case. Briefly, given two collections of solutions already found, that is, of collections $(\mathcal {P}',\mathcal {C'})$ of SPSs and SCSs, the algorithm finds a set $X\subseteq \mathcal {S}$ such that *X* does not contain any minimal SPS in $\mathcal {P}'$ and $\mathcal {S}\setminus X$ does not contain any minimal SCS in $\mathcal {C'}$ (or proves that such set does not exist). Since either *X* is a SPS or $\mathcal {S}\setminus X$ is a SCS, we have found a new solution not in $(\mathcal {P}',\mathcal {C'})$. Such a new solution is found in time $O(n(\tau +n))+m^{O(\log m)}$ where $n=|\mathcal {S}|$, *m* is the number of partial solutions already found (i.e. $m=|\mathcal {P}'| +|\mathcal {C}'|$) and $\tau$ is the time needed to evaluate *f*. Since $\tau$ is polynomial, we conclude the proof. $\square$

#### Relation to previous work

The paper in the literature that comes closest to ours is [8]. In fact, one of their definitions coincides completely with our definition of SPS. However, their work concentrates on a more restrictive model, which they call *machinery-duplicating*. The underlying idea of the latter is that each compound involved in a path from the precursor set to the target set should be produced in strictly positive amount, allowing a cell to therefore duplicate itself. We translate their definition by using the concept of factory (cf. Definition 2).

##### **Definition 7**

A *MD-stoichiometric factory* from $X \subseteq \mathcal {X}$ to $T \subseteq \mathcal {T}$ is a set $F \subseteq \mathcal {R}$, if there exists a flux vector $v \ge 0$ with $Y = {\textit{S}ubs(F)} \backslash X$ satisfying:

1. $$v_{i} \left\{ \begin{array}{ll} > 0 &{} \quad i \in F\\ = 0 &{} \quad\text {otherwise,} \end{array} \right.$$
2. $$(Sv)_{\mathcal {C}\setminus X} \quad \ge 0$$,
3. $$(Sv)_{T \cup Y} \quad > 0$$.

A set $X \subseteq \mathcal {X}$ is a *MD-stoichiometric precursor set* (MD-SPS) if there exists a MD-stoichiometric factory from *X* to $\mathcal {T}$.

Comparing this definition to Definition 2, clearly any MD-SPS is a SPS, but not the other way around. Moreover, not every minimal MD-SPS is a minimal SPS. In their work, the authors claim that for the growth of a colony of cells, one must consider that not only the biomass compounds should be produced in positive amount, but also all the reactants of every reaction with nonzero flux that are not sources. However, as we already mentioned, cycles like the one in Fig. 1 are considered unfeasible according to the machinery-duplicating model. Yet cycles with this structure are present in real networks and play an important role in metabolism, such as in the urea or the Krebs cycle.

### Enumerating precursor sets via MILP

In the following section, we describe how to enumerate all minimal $SPS$ and MD-SPS using a MILP approach similar to [10–12]. The authors of these papers describe methods that enumerate reaction subsets by recursively solving MILP problems. Therein, solutions obtained in a previous step are excluded from the solution space.

#### Enumeration of minimal $SPS$

We now present a practical method to enumerate all minimal stoichiometric precursor sets that allow to produce the set $\mathcal {T}$ in a positive amount. We iteratively solve a series of optimisation problems: at each iteration a mixed integer linear programming (MILP) problem is solved to obtain a minimal precursor set *X*; then we define a new MILP by adding a constraint that removes the obtained solution *X* and all the sets that contain it from the feasible set. We keep repeating this process until all solutions are found.

We need some additional definitions. For each source compound $x_j \in \mathcal {X}$, we add to $\mathcal {R}$ a reaction, which we call *source-pool reaction*, that produces $x_j$ from nothing (with stoichiometric coefficient 1). We denote this new set by $\overline{\mathcal {R}}$ and the set containing all source-pool reactions by $\overline{\mathcal {R}}_{\mathcal {X}}$. This set of reactions allows to model the availability of the source compounds since the upper bounds on their fluxes are linked to the amount of each source that is available. In the sequel $\overline{S}$ denotes the stoichiometric matrix *S* obtained by adding the columns given by the set of reactions $\overline{\mathcal {R}}_{\mathcal {X}}$ and *v* the flux vector, *U* is an upper bound constant for the values of each flux, $\epsilon$ is a vector of size $|\mathcal {T}|$ with an arbitrarily small positive real number in all coordinates, *b* is the vector of binary variables associated with each compound in $\mathcal {X}$, and we assume that $b_j=1$ ($b_j=0$) implies that compound $x_j$ is used (not used) to produce the target.

Given a network $\mathcal {N}=\left\{ \mathcal {C},\mathcal {R}\right\}$, a stoichiometric matrix *S*, a set $\mathcal {X}\subseteq \mathcal {C}$ of sources and a set $\mathcal {T}\subseteq \mathcal {C}$ of targets, we first define the following optimisation problem (1) to find *the first minimal solution*:

1 $$\begin{aligned} \begin{array}{cll} {\displaystyle \min f = } &{} {\displaystyle \sum _{j=1}^{|\overline{\mathcal {R}}_\mathcal {X}|} b_{j}}\\ s.t &{} \left( \overline{S}v\right) _{\mathcal {T}} \ge \epsilon , &{} \\ &{} \overline{S}v \ge 0 \\ &{} {\displaystyle b_{j} = 0 \leftrightarrow v_{j}= 0,} &{} \quad \forall j \in \overline{\mathcal {R}}_\mathcal {X}\\ &{} {\displaystyle b_{j} \in \left\{ 0,1\right\} ,} &{} \quad \forall j \in \overline{\mathcal {R}}_\mathcal {X}\\ &{} {\displaystyle 0 \le v_{i} \le U,} &{} \quad \forall i \in \overline{\mathcal {R}}\end{array} \end{aligned}$$

Model (1) is similar to the first MILP presented in [6]. The first set of constraints requires to produce the target at least in a quantity $\epsilon$. Instead of putting a small value for $\epsilon$ one could also put e.g. the maximum biomass yield. In this sense we enumerate all minimal precursor sets that allows for the maximal production of biomass. The third set of constraints (constraints ${\displaystyle b_{j} = 0 \leftrightarrow v_{j}= 0}$) denotes the fact that $v_j$—the flux associated to the source compound $x_j$—is positive if and only if $b_j=1$. These constraints can be formulated as a MILP as follows:

2 $$\begin{aligned} \left. \begin{array}{l} b_{j} \le v_{j} \\ v_{j} \le U b_{j} \end{array}\right\} \quad\text { for } \forall j \in \overline{\mathcal {R}}_\mathcal {X}, \end{aligned}$$

If $b_{j} = 1$, we have $v_{j} \ge 1$, which will force us to have at least one unity of the source compound *j*.

Since the objective function of (1) minimises $\sum _j b_j$, then the optimal solution $S^*$, is a precursor set of minimum cardinality. We now show how to modify the MILP to obtain all other minimal precursor sets. To this goal let the pair $(v^{*},b^{*})$ be an optimal solution to Problem (1). Let $I_{b^{*}}$ be the support of $b^{*}$; we consider the following constraint:

3 $$\begin{aligned} \sum _{j \in I_{b^{*}}} b_{j} \le |I_{b^{*}}| - 1, \end{aligned}$$

Constraint (3) excludes the solution $(v^{*},b^{*})$ and all the solutions that contain $b^{*}$ from the set of solutions of (1). Hence, adding to (1) constraints in the form (3) gives a new instance of the MILP whose solution is a new precursor set that is not included in the previously obtained ones and is minimal (though not necessarily of minimum cardinality). By repeating this procedure, which is a standard technique in mixed integer linear programming, we iteratively enumerate all minimal solutions.

If the obtained problem has no feasible solution, then we claim that we have found all minimal precursor sets.

#### MILP constraints for MD-SPS

In the work of Eker et al. [8], the machinery-duplicating model is defined through the use of linear constraints and boolean operators. If a set of sources is a MD-SPS, this implies it is a feasible solution according to their model. The authors also present a method to enumerate all MD-SPSs. Suppose we are given a set $\{X_1,\ldots , X_k\}$ of precursor sets that were already found. Their method consists in finding a minimal subset of sources *Y* that verifies two conditions: (1) *Y* has at least one source in common with each precursor set in $\{X_1,\ldots ,X_k\}$; and (2) the complement of *Y* must be able to produce the target according to the machinery-duplicating model. If one can find such a subset *Y*, a minimal precursor set can be obtained by taking the complement $\overline{Y}$ of *Y*, and finding one minimal subset of $\overline{Y}$. If no *Y* verifying the above conditions can be found, all minimal precursor sets have been enumerated and the algorithm stops.

Our method could also be adapted to consider the machinery-duplicating model presented by Eker et al.  [8]. The machinery-duplicating constraint is defined as:

4 $$\begin{aligned} \left( \overline{S}v\right) _{j} > 0 \vee \bigwedge _{i \in Q_{j}} v_i = 0, \end{aligned}$$

where $Q_{j}$ is the set of indices of reactions that use the compound *j* as a substrate. This can be reformulated into MILP constraints as:

5 $$\begin{aligned} \left( \overline{S}v\right) _{j} &\ge \overline{\epsilon }-D_{j} E_{j}, \\ \sum\limits _{i \in Q_{j}} v_i & \le D_{j} (1 -E_{j}), \end{aligned}$$

where $\overline{\epsilon }$ is an arbitrarily small positive real number, $D_{j}$ is a constant that can take any value greater or equal to $U|Q_{j}|$ and $E_{j}$ is an artificial binary variable. Adding (5) to Problem (1) allows us to enumerate all minimal stoichiometric precursor sets that respect the machinery-duplicating model.

## Results and discussion

In this section, we present the experiments we realised and discuss the results we obtained. We start by comparing the method we developed with the one of Eker et al. [8]. We then show the performance of sasita versus the approach where minimal $SPSs$ are obtained from combinations of minimal topological factories in the many-to-one network. Finally, we apply sasita to some genome-scale metabolic networks, obtained from Monk et al. [16]. The objective of this last part is both to illustrate how our method can be used and to validate it by reproducing the findings of the authors.

All the experiments were performed using an Intel QuadCore i7-4770 computer with 16 GB of RAM memory. The algorithm sasita is coded in Java (OpenJDK IcedTea) and uses cplex (IBM ILOG AMPL/CPLEX 12.5.1) for solving the MILP models; the constants are fixed as follows: $\epsilon = 0.5$, $\overline{\epsilon }= 0.5$, $U = 1000.0$. The constraints (2) were coded using indicator constraints to avoid numerical instability. The software and all network and input files can be downloaded at http://www.sasita.gforge.inria.fr.

### Comparison between sasita and Eker et al.’s approach

We start by calling attention to the fact that the comparison with the method of Eker et al. was difficult due to the fact that it is not publicly available. We also were not able to obtain it upon request. We therefore implemented a version of sasita that enumerates all minimal MD-SPS using the constraints given by Eq. (5). As input we took the metabolic network, the set of sources $\mathcal {X}$ and the set of targets $\mathcal {T}$ provided in the supplementary material of Eker et al. [8]. The authors provided also a list of “auxiliary compounds” without which, according to them, their model does not work. No auxiliary compound appears in the minimal precursor sets that are enumerated by Eker et al. It is not clear how these compounds are handled in their approach. If we treat such auxiliary compounds as ordinary ones, we are not able to enumerate a single MD-SPS with sasita. If we add a *source-pool reaction* for each one of the auxiliary compounds, we obtain the minimal MD-SPS X = {CCO-PERI-BAC@SULFATE}. Eker et al. find 787 solutions and all of them contain Sulfate. So the minimal solution *X* we found is in fact a subset of all their solutions.

We provide in the Additional file 1 a list of reactions *F* that form a MD-stoichiometric factory from *X* to $\mathcal {T}$, the flux values in *F*, and the stoichiometric matrix restricted to the reactions in *F*. Furthermore, we show that all substrates of the reactions in *F* and the target set $\mathcal {T}$ are produced in a positive amount using the reactions in *F*. Hence, the minimal MD-SPS fulfils the properties of a precursor set according to the machinery-duplicating model [8]. Such minimal MD-SPS is not found by Eker et al. probably because they do some preprocessing on the network that is not described in their paper and that we were not able to obtain upon request.

### Comparison between sasita and combinatorial approach

We ran both approaches, i.e. sasita and a combinatorial approach (called combi) where minimal $SPSs$ are obtained from combinations of minimal topological factories in the many-to-one network, on several instances. Our objective was to analyse the differences in the running times between both approaches, so we set cplex into single thread mode for sasita. Table 1 shows clearly that the MILP approach is more efficient than the combinatorial one. The networks of *S. muelleri*, *C. ruddii* and *B. aphidicola* were obtained from metexplore, filtering out ubiquitous metabolites and pairs of co-factors. We obtained the *E. coli* core model from http://www.systemsbiology.ucsd.edu/InSilicoOrganisms/Ecoli/EcoliSBML. As sources we considered all compounds that are not produced by a reaction or those that are produced by reversible reactions only. For the *E. coli* strains, we used the same networks from Monk et al. [16] and considered as sources the compounds from Table 3.

We set a time limit to the combinatorial approach of 2 h. sasita is by far more efficient on genome-scale networks where the combinatorial approach did not finish within the time limit. To be able to show an example where both approaches finish and sasita outperforms combi, we removed all compounds from the *E. coli* core network if they are consumed and produced by more than ten reactions. This is the case for M_atp_c, M_nad_c, M_nadh_c, M_h2o_c, M_h_e, M_h_c. The resulting network is denoted by a superscript a. Notice that the number of reactions remains the same because we remove only the above-mentioned compounds from the reactions. The difference in the time spent to solve the problem is remarkable. It takes less than 1 s with sasita and 42 s with combi.

**Table 1:** Our MILP approach (sasita) versus the combinatorial one

Strain	#Compounds/#reactions	#Sources/#targets	$t_{sasita}$ (s)	$t_{Combi}$ (s)
*S. muelleri*	76/64	9/Pyruvate	<1	<1
*C. ruddii*	128/126	45/Pyruvate	<1	1
*B. aphidicola*	282/245	91/l-Histidine	2	2
*E. coli* core^a^	72/126	14/Biomass core	<1	42
*E. coli* core	78/126	14/Biomass core	<1	*
*E. coli* CFT073	1911/2949	26/Biomass core	12	*
*E. coli* EDL993	1895/2943	25/Biomass core	13	*
*E. coli* K-12	1806/2854	25/Biomass core	12	*
*E. coli* Sakai	1895/2942	25/Biomass core	12	*

^a^ Filtered network (see text). The MILP approach (sasita) and the combinatorial one (combi) are applied to several metabolic networks. For each instance, we provide the size of the network (number of compounds and reactions), the number of source and target compounds, and the time spent by both approaches ($t_{sasita},\ t_{Combi}$). One asterisk means that the combinatorial approach could not finish within the time limit

### Enumerating minimal precursor sets in genome-scale metabolic networks

In this case, we based our experiments on the work of Monk et al. [16] who investigated the pan and core metabolic capabilities of 55 *Escherichia coli* and *Shigella* strains based on genome-scale reconstructions of their metabolism. By core is meant the elements shared by all strains and by pan the union of the elements from all strains. As concerns the latter in particular, the authors found the pan to be enriched in alternate carbon metabolic pathways. In order to determine the functional differences among the strains, the authors computed by flux balance analysis (FBA) the growth phenotypes of 385 nutrients (henceforth called the *test metabolites/compounds*), each considered individually as a source of carbon, nitrogen, phosphorus and sulfur, aerobically and anaerobically. To that purpose, an in silico minimal medium that contains a sole carbon, nitrogen, phosphorus and sulfur source was defined. The authors then replaced the sole carbon source by each of the 385 test metabolites one at a time. Whether or not these new media constituted a growth condition was tested by FBA. The procedure was repeated for each source in the minimal media, namely for nitrogen, phosphorus and sulfur, as well as for each strain. The resulting metabolic phenotypes indicated strain-specific adaptation to nutritional environments.

Our first goal was to validate our method: we compared the results obtained with sasita to the ones in Monk et al. [16]. We enumerated and compared the minimal precursor sets allowing for biomass production of the *E. coli* strains, which included commensals as well as both intestinal and extraintestinal pathogens. We used for this the genome-scale metabolic models from Monk et al. [16]. The strains were *E. coli* str. K-12 MG1655 (Commensal), *E. coli* O157:H7 str. Sakai (Enterohemorrhagic *E. coli*, EHEC), *E. coli* O157:H7 EDL933 (EHEC), and *E. coli* CFT073 (Uropathogenic *E. coli*, UPEC). The same 385 compounds tested in [16] were given as part of the sources for different runs of sasita. Since Monk et al. [16] were not interested in minimal solutions, we wanted to check whether our solutions were subsets of their solutions.

The second goal was to explore some solutions that were only found by sasita in order to illustrate one application of our method. These solutions contain more than one of the test metabolites, after excluding sources from the minimal media (i.e. carbon, nitrogen, phosphorus and sulfur). Such solutions were explored as concerns strain-specific growth and their relation to niches and to pathotypes.

For almost all solutions found by Monk et al. [16], sasita was capable of correctly finding at least one corresponding minimal subset. There is a small number of solutions (7) found by Monk et al. [16] and not by sasita. In Table 2, we show the amounts of solutions found and not found for each strain. We confirmed through FBA that there is indeed no feasible flux for those solutions (this is further discussed below). In the remainder of this section, we explain how we realised our experiments and we present our results in more detail.

**Table 2:** Differences between solutions found by Monk et al. [16] and by sasita

Network	Matches	Not found
*E. coli* CFT073	599	0
*E. coli* O157:H7 EDL933	597	0
*E. coli* str. K-12 MG1655	607	7
*E. coli* O157:H7 str. Sakai	597	0

The column “Matches” has the amount of solutions from [16] for which we found at least one subset. The column “Not found” indicates the amount of solutions from [16] for which we could not find a correspondence

Two experiments were conducted. In both cases, oxygen was always available and we used as target an artificial compound that is added as an extra product of the core biomass reaction, with stoichiometry of 1.0. Also, the minimal media for *E. coli* CFT073 contained tryptophan, its auxotrophy. We now give a general description of each experiment. The exact list of compounds for each experiment as well as all networks can be found in the sasita website.

In Experiment 1, we tested growth on minimal media of each strain as defined by Monk et al. [16] by enumerating the minimal precursor sets using as sources only the compounds from such minimal media. In Table 3, we present the list of compounds considered as sources for each strain, for this experiment. For each strain we found two solutions, one aerobic and another anaerobic as expected. This shows that, theoretically, the so-called “minimal media” are in fact not minimal as a whole (i.e. they are minimal in terms of carbon, sulfur, nitrogen and phosphorus sources) and that the considered strains can grow from a proper subset of that set of compounds.

**Table 3:** Minimal media compounds for each *E. coli* strain

Strain	Minimal media
*E. coli* CFT073	Calcium, cob(I)alamin, chloride, $\text{Co}^{2+}$, $\text{Cu}^{2+}$, $\text{Fe}^{2+}$, $\text{Fe}^{3+}$, $\text{H}^{+}$, potassium, magnesium, $\text{Mn}^{2+}$, molybdate, calcium, nickel, selenate, selenite, tungstate, zinc, 6-Acetyl-d-Glucose, sulfate, ammonium, diphosphate, nicotinate, l-Tryptophan, $\text{O}_{2}$
*E. coli* EDL993 , K-12, Sakai	Calcium, cob(I)alamin, chloride, $\text{Co}^{2+}$, $\text{Cu}^{2+}$, $\text{Fe}^{2+}$, $\text{Fe}^{3+}$, $\text{H}^{+}$, potassium, magnesium, $\text{Mn}^{2+}$, molybdate, calcium, nickel, selenate, selenite, tungstate, zinc, 6-Acetyl-d-Glucose, sulfate, ammonium, diphosphate, nicotinate, $\text{O}_{2}$

In order to check the ability of each strain to grow on the 385 test compounds as sources of carbon, nitrogen, phosphorus and sulfur, we ran Experiment 2. For each network we considered as input source set a subset of the minimal media compounds plus a subset of the 385 test metabolites.

For subsets of the minimal media compounds, we considered the set of minimal media compounds minus one of the following: glucose, ammonium, phosphate or sulfate respectively. Since we were removing a compound from the minimal media, we included only the test metabolites that could replace the removed one (if we removed glucose, we considered only the set of test compounds that have carbon in their composition and so on). This was done because considering all the 385 test metabolites together leads to a combinatorial explosion of the number of solutions that are unpractical to enumerate with sasita. In one case, namely when we remove glucose, the set of test metabolites to include was also too big and we needed to split it further in two smaller sets. As a side effect, this split of the input compounds can lead to a loss of some solutions, namely those containing compounds that are in different input sets. We thus may lose some solutions that have more than one minimal media compound replaced by two or more test metabolites. However, our split guarantees that at least all the sets considered in Monk et al. [16] are possible combinations of our input compounds because the authors replace glucose, ammonium, phosphate or sulfate from the minimal media with only one of the test metabolites.

*First goal: Comparison with * Monk et al. We found 837 minimal precursor sets for *E. coli* CFT073 and between 11.164 and 13.732 for the other strains (Table 4).

**Table 4:** Number of solutions found for each *E. coli* strain

Strain	Solutions
*E. coli* CFT073	837
*E. coli* EDL993	11.164
*E. coli* K-12	13.732
*E. coli* Sakai	11.164

This difference is remarkable but not surprising: *E. coli* CFT073 has a tryptophan auxotrophy, and tryptophan itself can be a source of carbon and nitrogen. Since we search for minimal precursor sets and tryptophan is always a source, there is no need for any extra source of carbon and nitrogen, thus reducing the number of solutions for this strain.

All solutions found by sasita are either a subset of the given minimal media or a subset of the minimal media plus one or more test metabolites. The number of solutions where at least one of the four sources (carbon, nitrogen, phosphorus and sulfur) is replaced by only one compound among the 385 test metabolites is presented in Table 5.

**Table 5:** Number of solutions with one test metabolite

Sources	*E. coli* CFT 073		*E. coli* EDL933		*E. coli* K-12		*E. coli* Sakai	
	$\text {O}_{2}$	No $\text {O}_{2}$	$\text {O}_{2}$	No $\text {O}_{2}$	$\text {O}_{2}$	No $\text {O}_{2}$	$\text {O}_{2}$	No $\text {O}_{2}$
C	0	0	104	51	109	61	104	54
C, N	0	0	51	42	52	44	51	42
C, N, P	0	0	37	22	38	22	37	22
C, N, S	0	0	8	0	8	0	8	0
C, P	0	0	14	22	13	21	14	22
C, S	0	0	0	0	2	0	0	0
N	0	0	11	14	14	10	11	14
P	51	51	6	6	7	7	6	6
S	22	0	14	0	10	0	14	0
Minimal media	1	1	1	1	1	1	1	1
Total	74	52	246	158	254	166	246	161
Total	126		404		420		407	

Number of solutions with one test metabolite and removing one or more sources from the minimal media, namely carbon (C), nitrogen (N), sulfur (S) or phosphorus (P)

These solutions contain one test metabolite plus a subset of the minimal media in which the test metabolite replaces one, or more of the following compounds: glucose, ammonium, phosphate and sulfate. They therefore correspond to minimal sets of the solutions found by Monk et al. [16].

Other differences found in the comparison of our results with those from [16] are presented and discussed below. Some such differences arise for *E. coli* CFT073 for which, as mentioned, tryptophan can always be a source of carbon and nitrogen. Since we find a minimal solution without any test metabolite and without glucose and ammonium, but with tryptophan, it is a minimal subset of all solutions from [16] considering the replacement of glucose or ammonium by a test metabolite. Furthermore, a few minimal precursor sets for which Monk et al. [16] found no growth are present among our solutions because of the different conditions we allowed in our test, namely some sources were available at bigger amounts and the compounds were allowed to accumulate. There were as well seven solutions for which Monk et al. [16] found a positive flux and we did not (see Table 2). Those solutions are for the K-12 strain. Among these solutions, 6 are related with the compounds 4-Hydroxy-l-threonine and Oxaloacetate from the exchange subsystem, and in fact there are no reactions in the network that use those compounds to produce anything. The remaining solution is the one using Thiosulfate as source of sulfur, and we confirmed no growth by FBA for this condition. There is one last difference, for the solution with the test metabolite Fe(III) dicitrate which allows growth as a carbon source in aerobic and anaerobic conditions for *E. coli* K-12. We explicitly found only the anaerobic solution. Since the aerobic one can be seen as a superset of the anaerobic, it is not a minimal precursor set. This does not happen in the other test metabolites owing to different iron oxidation states in each solution. We thus found different aerobic and anaerobic solutions when we enumerated the minimal precursor sets.

In conclusion, we obtained almost all of the solutions found by Monk et al. [16], showing that the nutrient sources of alternate catabolic pathways are part of the minimal precursor sets that allow for biomass production in most of the tested *E. coli* strains.

*Second goal: Original results using*sasita

The remaining solutions go beyond the analyses performed by Monk et al. [16] because they contain two or more test metabolites (Table 6).

**Table 6:** Solutions with more than one test metabolite

Test metabolites	Solutions	*E. coli* strains
2	710	CFT073
2	3.387	EDL993;K-12;Sakai
2	298	EDL993;Sakai
2	656	K-12
3	4.992	EDL993;K-12;Sakai
3	703	EDL993;Sakai
3	2.106	K-12
4	1.027	EDL993;K-12;Sakai
4	299	EDL993;Sakai
4	1.113	K-12
5	19	EDL993;K-12;Sakai
5	21	EDL993;Sakai
5	5	K-12
6	5	EDL993;K-12;Sakai
6	4	EDL993;Sakai

Most of these solutions actually have two or three test metabolites (4.961 and 7.801 respectively). In both cases, more than 60 % of those solutions are aerobic.

Most solutions in which the two or three test metabolites replace all four sources from the minimal media (glucose, ammonium, phosphate, sulfate) are found in all the three strains, *E. coli* EDL993, *E. coli* K-12 and *E. coli* Sakai. Moreover, there are some minimal precursor sets specific to *E. coli* EDL993 and *E. coli* Sakai which are both EHEC, and others specific to *E. coli* K-12 which is commensal (Table 7). The latter are specific to pathotypes and probably indicate adaptations to nutritional environments.

**Table 7:** Solutions with two or three test metabolites

Test metabolites	Solutions	*E. coli* strains
2	674	CFT073
2	556	EDL993; K-12; Sakai
*2*	*6*	*EDL993; Sakai*
3	2.641	EDL993; K-12; Sakai
3	143	EDL993; Sakai
3	511	K-12

Solutions with two or three test metabolites without any of the four sources from the minimal media (carbon, nitrogen, phosphorus and sulfur). Further details about the row in italic is given in the text

From these results, the pairs of test metabolites in the six solutions specific to *E. coli* EDL993 and *E. coli* Sakai are: *N*-Acetyl-d-galactosamine 1-phosphate with butanesulfonate, ethanesulfonate or taurine, respectively; all aerobic and each one in two solutions with different iron states (line in italics in Table 7). *N*-Acetyl-d-galactosamine 1-phosphate was shown to give extraintestinal pathogenic strains of *E. coli* a catabolic advantage when compared to commensals, supporting growth in 100 % of the cases compared to 67 %, respectively [16]. This compound supported growth as a sole carbon source in aerobic and anaerobic conditions for extra and intracellular pathogens (*E. coli* CTF073 together with tryptophan, *E. coli* EDL993 and *E. coli* Sakai), however not for the commensal strain *E. coli* K-12. Furthermore, the enterohemorrhagic strains *E. coli* EDL933 and *E. coli* Sakai were shown to occupy the same niche in the streptomycin-treated mouse intestine [17] while *E. coli* EDL933 was shown not to colonise the same niche and does not use the same sugars as carbon source as the commensal *E. coli* K-12 [18, 19]. The three solutions detailed above and the solutions presented in Table 7 therefore represent metabolic capabilities that are specific to the pathogenic strains analysed here when compared to the commensal strain *E. coli* K-12, in agreement with the niches occupied by such strains.

These results suggest that sasita can depict pathotype and niche-specific metabolic capabilities which allow broad in silico studies of strains or species interactions. For instance, an extension of the analysis presented in this paper to a larger dataset of *E. coli* strains including both pathogenic and commensal biotypes could help predict in silico sets of commensal strains that would prevent the colonisation of pathogens due to a consumption by the native microbiota of the nutrients required by the pathogen (see the experimental study of mutant phenotypes in Maltby et al. [20]).

## Conclusions

We examined the relationship between topological and stoichiometric precursor sets. We highlighted that stoichiometric precursor sets can be obtained from combinations of minimal topological factories in the many-to-one network. However, this does not lead to an efficient method. We then presented sasita, an efficient algorithm for the exhaustive enumeration of minimal precursor sets for a given target that takes into account stoichiometry. To the best of our knowledge, there exists only one previous approach for this problem due to Eker et al. [8] who proposed two different constraint models, steady-state and machinery-duplicating. However, in their computations, the authors use only the latter, that requires a strictly positive net production of the intermediate compounds on the path from the sources to the target. This model may exclude solutions as we showed (Fig. 1).

In our experiments, we enumerated and compared the minimal precursor sets of nutrient sources of alternate catabolic pathways allowing for biomass production of some *Escherichia coli* strains, comprising commensal and both intestinal and extraintestinal pathogens, using genome-scale metabolic models. We compared our results to those of Monk et al. [16] in order to have a guideline on part of the solutions we generated, since our approach is different from the one that the authors used, and our results go beyond such comparison. We found metabolic capabilities that distinguish the strains compared in their ability to catabolise nutrients, and such were specific to pathotypes and niches of *E. coli* strains.

Our method can therefore be used in a wide variety of applications in order to study minimal growth conditions as well as strains and/or species interactions based on their catabolic abilities and their nutritional niches. One valuable application in this context would be to predict patterns of colonisation of commensal and pathogenic *E. coli* strains in the intestine.

Our method can furthermore be used to refine a metabolic network. If growth of an organism is observed for a defined medium in the laboratory, but no minimal precursor set is a subset of such medium, then either the metabolic network lacks reactions, e.g. export reactions, or the biomass function is not well formulated.

The execution of all experiments of the comparison with Monk et al. [16] took altogether around 5 days. The execution times ranged from 20 s to 12 h. Running the experiments in parallel, one could such retrieve the results after less than a day. We cannot claim to be more efficient than Eker et al. [8] as their software is not available for testing. However, we are guaranteed to enumerate all minimal $SPSs$ and MD-SPS.

## Additional file

10.1186/s13015-016-0087-3 A list of reactions *F* that form a MD-stoichiometric factory, the stoichiometric matrix restricted to the reactions in *F*, one possible flux vector *v*, and the vector of the net production of all compounds.
